# A Rare Case of Giant Meckel’s Diverticulum in an Adult: Diagnostic and Therapeutic Challenges

**DOI:** 10.7759/cureus.107543

**Published:** 2026-04-22

**Authors:** John Nobles, Frances Morana, Genevieve Crawley, Reginald Jones

**Affiliations:** 1 General Surgery, California Hospital Medical Center, Los Angeles, USA; 2 School of Medicine, Ross University School of Medicine, Bridgetown, BRB; 3 General and Trauma Surgery, California Hospital Medical Center, Los Angeles, USA

**Keywords:** complicated meckel’s diverticulum, gastrointestinal surgery, giant meckel’s diverticulum, meckel's diverticulum, meckel's diverticulum in adults

## Abstract

Meckel's diverticulum is the most common congenital anomaly of the gastrointestinal tract, resulting from incomplete obliteration of the vitelline duct. Typically located within 2 feet of the ileocecal valve, it occurs in approximately 2% of the population. While often asymptomatic and most commonly diagnosed in pediatric patients, Meckel's diverticulum in adults is rare and frequently presents with complications such as obstruction, inflammation, ischemia, or perforation. Its non-specific clinical presentation in adults can make diagnosis challenging and often delays definitive management until surgical exploration is performed. Oftentimes, imaging can be non-specific; as in this case, it supported a mesenteric cyst more than a Meckel’s diverticulum. This report highlights a case of a 54-year-old male who presented with vague abdominal symptoms and was subsequently diagnosed through imaging and successfully treated with surgical removal of a 12×11×8 cm giant Meckel’s diverticulum, defined as any diverticulum larger than 5 cm. This report aimed to increase clinical awareness of Meckel's diverticulum in adults and reinforce the need for early detection and prompt surgical intervention in complicated cases.

## Introduction

Meckel's diverticulum is a congenital defect arising from incomplete obliteration of the vitelline duct, typically located in the distal ileum. Meckel's diverticulum is one of the most common congenital gastrointestinal malformations, presenting in approximately 2% of the general population. According to the "rule of 2's," Meckel's diverticulum is typically located within 2 feet of the ileocecal valve, usually measures approximately 2 inches in length, may contain two types of heterotopic mucosa, often gastric or pancreatic tissue, and is most commonly diagnosed in patients under two years of age [1]. However, while Meckel's diverticulum is often asymptomatic, it can present with various complications, particularly in adults, which may delay diagnosis and management [2]. While most Meckel's diverticula are under 5 cm, giant Meckel's diverticulum variants exceed 5 cm, with some reported as even over 10 cm.

Although Meckel's diverticulum is frequently observed in pediatric populations, adult presentations are more likely to involve complications such as intestinal obstruction, diverticulitis, perforation, or, rarely, malignancy [3,4]. Giant Meckel's diverticulum, which exceeds the typical size and is a rare but serious cause of intestinal obstruction, may also lead to torsion or necrosis, further complicating diagnosis and treatment [5,6]. The location and size of the diverticulum govern the clinical presentation and the need for surgical intervention [7].

The diagnostic challenge in Meckel's diverticulum lies in its varied and often non-specific clinical manifestations that can stay clinically silent until it presents as a surgical emergency or develops into a malignancy. Moreover, a common misconception is that Meckel's diverticulum primarily affects the pediatric population, which may lead to its underdiagnosis in adults. Imaging modalities such as CT angiography (CTA), multiphase CT enterography, ultrasound, MRI, and nuclear medicine are essential for detecting Meckel's diverticulum, particularly when presenting with vague, unexplained gastrointestinal symptoms [7,8]. Tc-99m pertechnetate scans are still used in pediatric patients, but their sensitivity and usefulness can vary, with higher sensitivity in pediatric populations (85-90%) than in adult patients (60%), and are more useful when the Meckel’s diverticulum contains gastric tissue [7].

The mainstay of treatment for pediatric asymptomatic and pediatric and adult symptomatic Meckel’s diverticulum is surgical management, while treatment of asymptomatic adult patients remains debated, with a preferential conservative, non-surgical approach involving monitoring, bowel rest, and antibiotics infrequently.

The following two main surgical approaches are often used for symptomatic Meckel’s diverticulum management: diverticulectomy and wedge or segmental resection. Which procedure is recommended is often based on the integrity of the diverticulum base and adjacent ileum, as well as the location and presence of ectopic tissue. The location and presence of ectopic tissue cannot always be determined accurately; therefore, its position is often estimated based on the height-to-diameter ratio. Long diverticula (>2.0) tend to have ectopic tissue located away from the base, whereas short diverticula (<2.0) tend to have ectopic tissue closer to the base [6].

A diverticulectomy is suggested for simple symptomatic Meckel’s diverticulum of a long diverticulum, while wedge and segmental resection are recommended for short diverticula, complicated intestinal obstruction, complications with bleeding, inflamed or perforated base, and tumor [6]. This case illustrates an adult presentation of a giant Meckel's diverticulum and highlights the challenges associated with this condition.

## Case presentation

**Figure 1:**
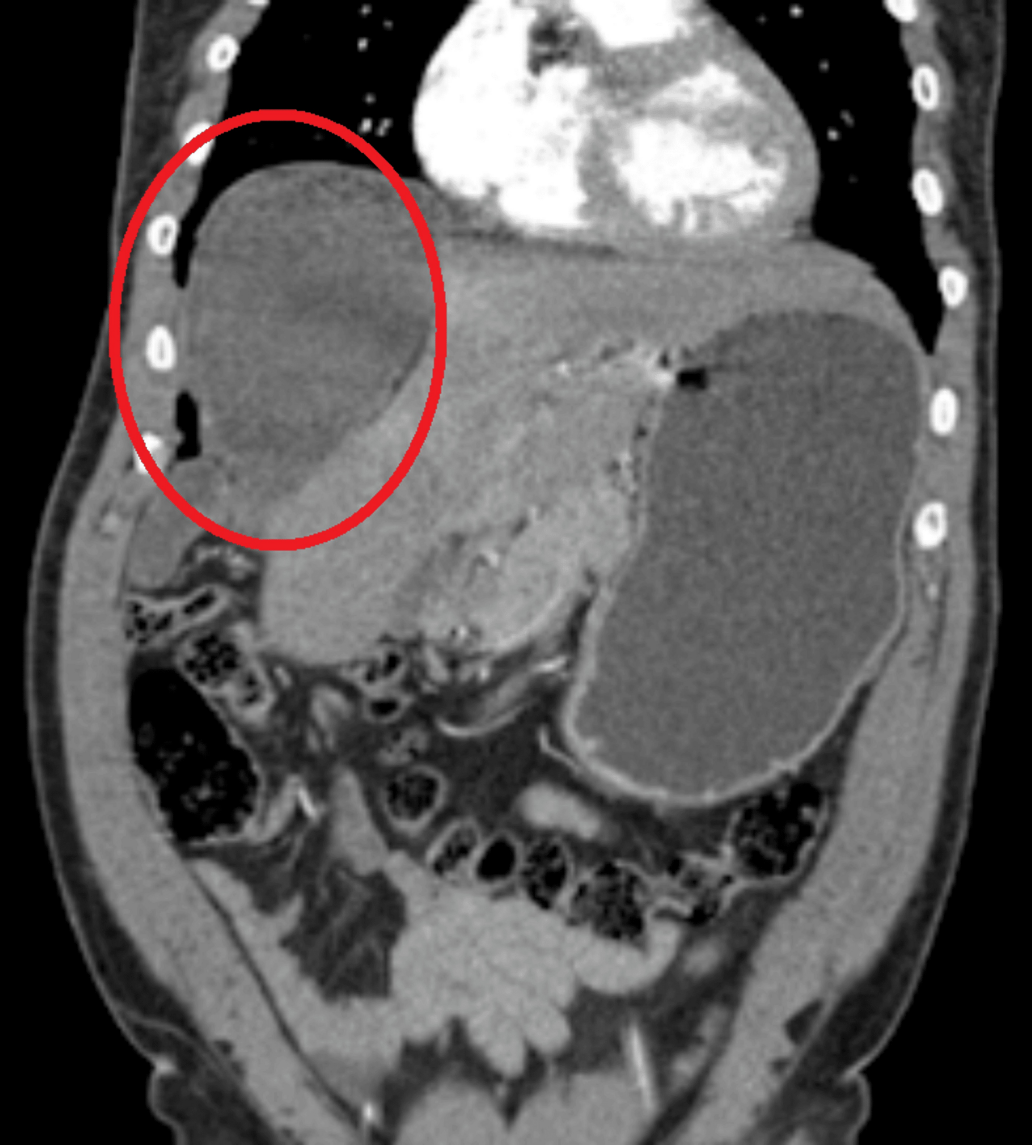
Initial CT angiography with contrast, coronal view. There is a well-circumscribed, rounded structure in the right upper quadrant (circled in red) with predominantly fluid attenuation. A large, well-circumscribed, low-attenuation cystic lesion occupying the left upper quadrant of the abdomen, likely arising from or abutting the stomach. The lesion demonstrates homogeneous fluid attenuation without internal septations, mural nodularity, or obvious solid enhancing components. There is significant mass effect with displacement of adjacent structures, including inferior displacement of bowel loops and medial deviation of surrounding viscera. The stomach appears compressed and displaced, suggesting either an exophytic gastric origin or close association with the gastric wall. No obvious pneumoperitoneum. No free fluid is definitively identified on this image. The visualized bowel loops are non-dilated without clear obstruction.

**Figure 2:**
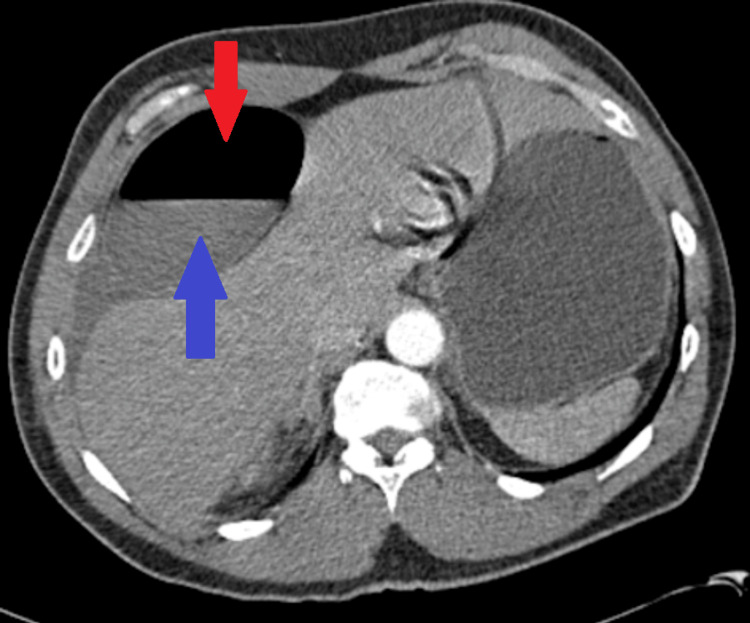
Initial CT angiography with contrast, axial view. The portion marked with the red arrow demonstrates a large intraluminal gas collection, suggesting a segment of bowel or gas within a cystic structure. The portion marked with the blue arrow shows dependent fluid, consistent with a fluid-fluid level within the same structure. There is a large, well-defined, round to ovoid low-attenuation cystic lesion in the left upper quadrant, measuring several centimeters in maximal diameter. The lesion demonstrates homogeneous fluid attenuation without internal septations, calcifications, or enhancing solid components. The wall appears thin and smooth. The lesion exerts significant mass effect as follows: medial displacement of the stomach, posterior displacement of adjacent structures, compression of surrounding bowel loops, and no clear attachment to any other structures. No surrounding inflammatory stranding is seen. No free intraperitoneal air. No appreciable ascites.

The patient is a 54-year-old male with a past medical history (PMH) of uncontrolled hypertension (HTN), hyperlipidemia (HLD), and peripheral artery disease (PAD) who initially presented to the emergency room at California Hospital Medical Center with complaints of vague abdominal pain, nausea, and vomiting (N/V), and reported that he had never experienced this pain previously. A CT abdomen and pelvis (CTAP) with contrast showed a fluid-filled structure containing an air-fluid level and small calcifications within the right upper abdomen, suggestive of a possible Meckel's diverticulum or mesenteric cyst (Figures 1, 2). The patient had an elevated BP of 215/97 mmHg and admitted to not having taken his blood pressure medication for a few weeks. The patient was complaining of diffuse abdominal pain that extended from his lower abdomen to his upper abdomen and into his chest. On physical examination, abdominal findings were as follows: soft abdomen, normal bowel sounds, diffuse abdominal tenderness to palpation, and no guarding or rebound. White blood cell count was 8.9×10³/μL; glucose was 143 mg/dL; the patient was afebrile (36.7°C); heart rate was 55 bpm; and no lactate level was measured at that time. An EKG was performed and showed no ST changes and was within normal limits. The patient's condition seemed to improve with symptomatic relief, and vitals improved with treatment. Cardiac concerns, such as aortic dissection and myocardial infarction, were ruled out, with no evident signs of bowel obstruction at that time and imaging suggestive of normally benign lesions such as a mesenteric cyst or Meckel's diverticulum. The patient was subsequently discharged for outpatient care and removal of the potential diverticulum or cyst. However, the patient returned to the emergency department within 24 h for persistent abdominal pain.

**Figure 3:**
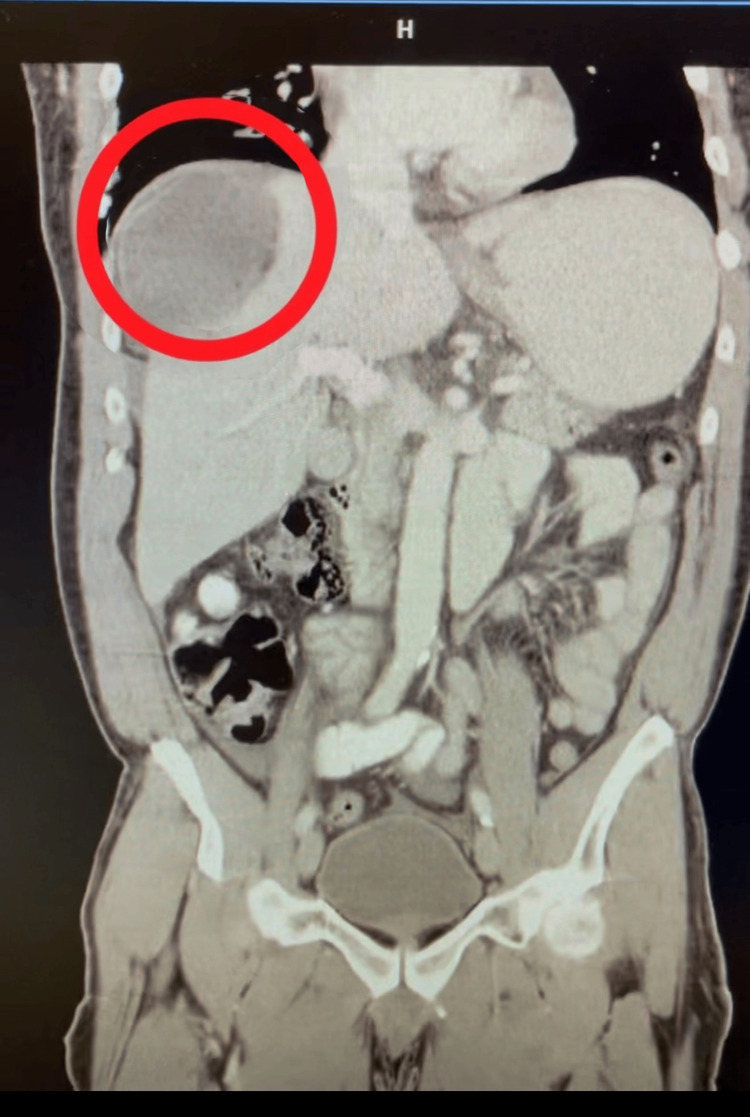
Repeat CT scan of the abdomen and pelvis in coronal view. There is a well-circumscribed, rounded structure in the right upper quadrant (circled in red) with predominantly fluid attenuation. A small amount of intraluminal gas is seen within the superior aspect of the structure, suggesting communication with bowel or entrapped air. The lesion appears to displace adjacent small bowel loops inferiorly, consistent with a localized mass effect. Surrounding peritoneal fluid is noted, suggestive of reactive or inflammatory changes. No evidence of free intraperitoneal air is identified. Liver, spleen, pancreas, and kidneys appear unremarkable in this section.

**Figure 4:**
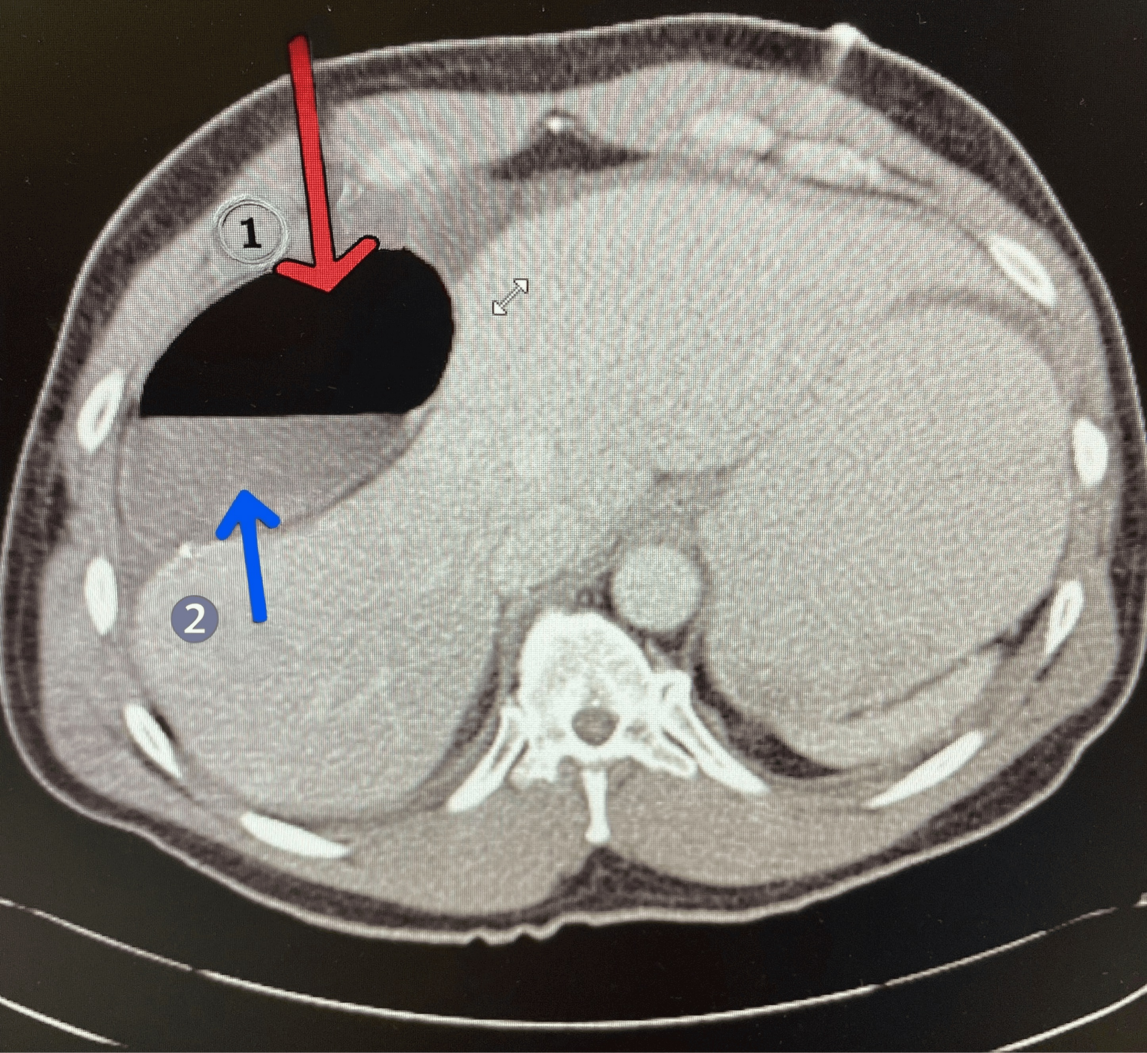
Repeat CT of the abdomen and pelvis in axial view. There is a well-defined, predominantly fluid-attenuation structure in the right upper quadrant, anterior to the liver, containing both hypodense (fluid) and hyperdense (air) components. The portion marked with the red arrow demonstrates a large intraluminal gas collection, suggesting a segment of bowel or gas within a cystic structure. The portion marked with the blue arrow shows dependent fluid, consistent with a fluid-fluid level within the same structure. There is surrounding free fluid within the peritoneal cavity. No obvious evidence of pneumoperitoneum or portal venous gas is identified. The liver, spleen, pancreas, and kidneys appear unremarkable in this section.

A repeat CTAP with contrast demonstrated findings concerning a possible closed-loop small-bowel obstruction located in the right upper quadrant, anterior to the liver (Figure 3). There was an increase in free fluid throughout the abdomen and pelvis compared to the previous study, raising concern for early bowel ischemia (Figure 4). No evidence of pneumatosis, portal venous gas, or perforation was noted. A surgical consultation was recommended. In the interim, the patient was started on intravenous fluids, empiric antibiotics, and pain management. Despite initial treatment, he continued to experience diffuse abdominal pain, with similar physical examination findings as the day before, a heart rate around 73 bpm, leukocytosis with a white blood cell count of 20.2×10³/μL, and a lactic acid level of 2.1 mmol/L.

**Figure 5:**
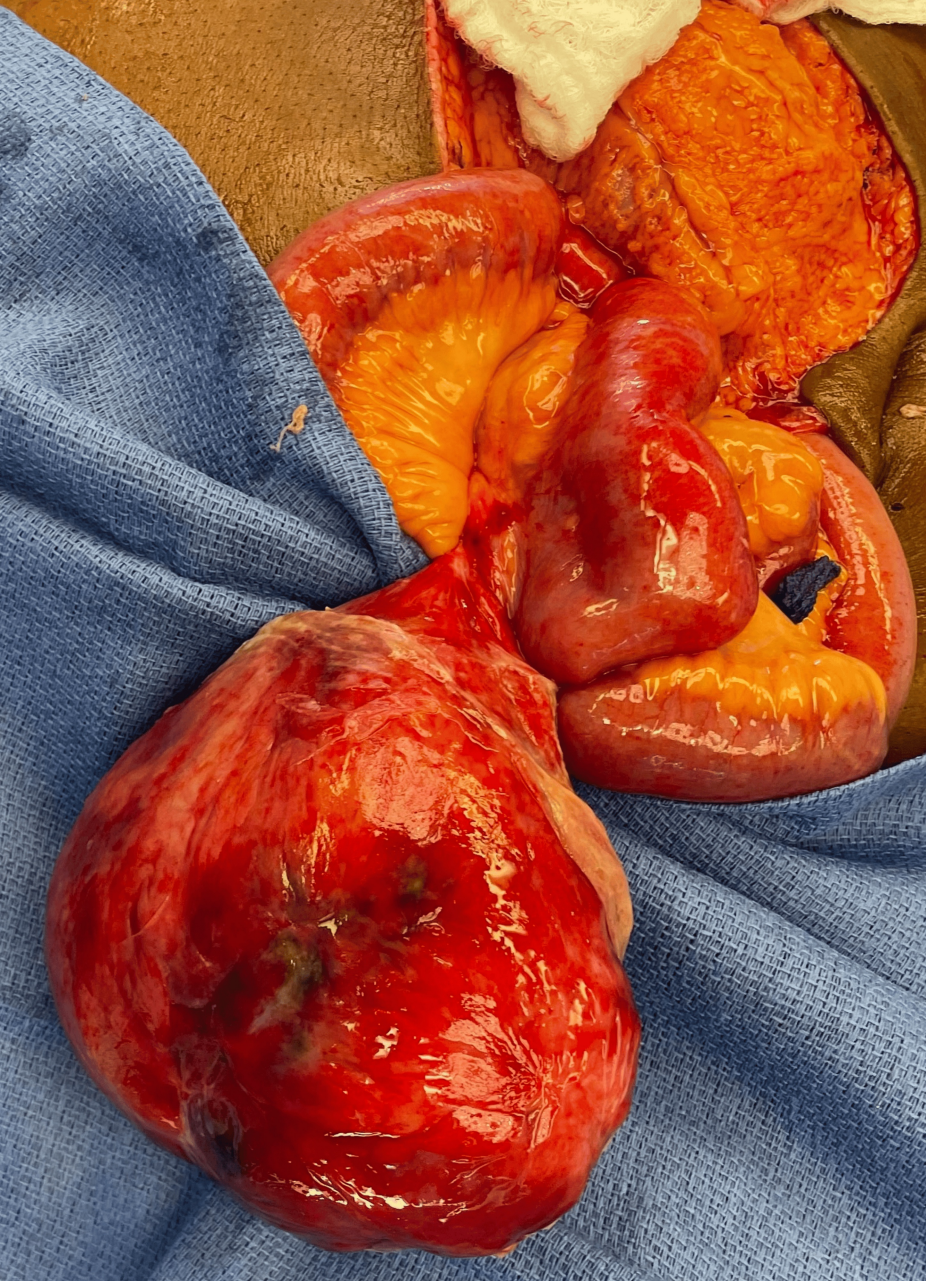
Gross appearance of the giant Meckel’s diverticulum at the time of surgery. This giant Meckel's diverticulum was measured to be around 12x11x8 cm with a narrow base.

Given his worsening clinical status and imaging findings, the patient was taken to the operating room for an exploratory laparotomy. Intraoperatively, a midline laparotomy incision was made down to just below the umbilicus, and the abdominal cavity was entered through the fascia. Inside the abdominal cavity, there was a significant amount of turbid fluid, which was evacuated, and a wide local exposure was established using a Bookwalter retractor (Nashville, Tennessee: Symmetry Surgical). The small bowel was examined from the ligament of Treitz toward the ileum, where there was tethering of the bowel in the right upper quadrant. The tethered distal ileum had a mass consistent with a large Meckel's diverticulum encased in purulence and fibrinous exudate. The mass was elevated from the cavity, causing the release of fibrinous exudate. The mass was an extension from the distal ileum, approximately 2 feet from the ileocecal valve, and had a narrow base. Using an Endo GIA (Dublin, Ireland: Medtronic), the mass was excised in a transverse fashion, and the staple line was oversewn with 3-0 GI silk in an inverted Lembert fashion. The mass was sent to pathology. The cavity was thoroughly irrigated with 3 L of saline (Figure 5). Through a separate incision, a 19-round Blake drain was placed in the space left after the diverticulum was removed to monitor postoperative output. The midline incision was closed using a #1 looped polydioxanone (PDS) suture, starting at the subdermal superior aspect and closing in a continuous fashion transversely in the pelvis. The patient remained under close monitoring by the surgical team over the following five days.

The pathology report showed that the specimen consisted of a roughly spherical 12×11×8 cm cyst-like structure with a glistening, dark green surface, soft, fibrous serosal adhesions, and a 4 cm-long pedicle closed with surgical staples. Sectioning revealed a cystic structure with a 0.5 cm-thick wall. The lining was mottled brown-gray and dark brown. On microscopic examination, sections showed a wall composed of a well-defined muscle layer overlying fat of varying thickness. The lining consisted of fibrinous and acute inflammatory cell exudate. No mucosa was identified, most likely due to the infarction and necrosis of the Meckel's diverticulum. These findings were most consistent with an obstructed Meckel's diverticulum with marked acute inflammation and necrosis.

By postoperative day (POD) five, the patient denied nausea, vomiting, or abdominal pain. Patient tolerated a regular diet, passed flatus and bowel movements, was voiding normally, and ambulating without difficulty. The JP drain was also removed on POD five, and the patient was cleared for discharge. Follow-up with the patient at four and eight weeks postoperatively showed that he was feeling well, with no complaints at both visits.

## Discussion

This case highlights the diagnostic complexity and clinical significance of Meckel's diverticulum in adult patients, particularly when presenting with acute abdominal symptoms. Although Meckel's diverticulum is commonly associated with pediatric populations, this case reinforces that it must not be overlooked in adults presenting with signs of small bowel obstruction or abdominal sepsis of unclear etiology. The patient's initial presentation with abdominal pain, nausea, and vomiting, accompanied by CT imaging suggesting a mesenteric cyst or a Meckel’s diverticulum, did not raise urgent surgical consultation, as the patient was given an outpatient referral for further workup. The delay in definitive diagnosis is consistent with the literature, which emphasizes the non-specific and often misleading clinical manifestations of symptomatic Meckel's diverticulum in adults [2,3]. His return to the emergency department with persistent symptoms and subsequent imaging indicating a closed-loop obstruction with increasing free fluid raised concern for evolving bowel ischemia, prompting urgent surgical evaluation. Intraoperative findings revealed a large, gangrenous Meckel's diverticulum with fibrinous exudate, consistent with advanced inflammation and compromised perfusion. The atypical size complicated preoperative identification, fitting the profile of a giant Meckel's diverticulum, which has been associated with an increased risk of torsion, strangulation, and necrosis [5,6].

Reported sizes for Meckel’s diverticulum vary greatly, from 1 to 56 cm in length and from 1 to 50 cm in diameter. Some limited surgical studies suggest that there may be heterotopic tissue in 57% of symptomatic Meckel’s diverticula, while a 6% incidence of heterotopic tissue was found in autopsy specimens of asymptomatic diverticula [9]. This large variation in size and the potential presence of heterotopic tissue can make identifying Meckel’s diverticulum more difficult and can lead to various complications. Adults with Meckel’s diverticulum have obstruction in 35.6% of cases, hemorrhage in 27.3%, and inflammation in 29.4% [7]. Longer diverticula and those with narrow bases, such as in this case, are more prone to torsion, ischemia, and necrosis, as the length and narrow base allow for greater rotation of the diverticulum and for it to swing like a pendulum, which can easily compromise the often incomplete or poorly developed blood supply.

These complication risks demand heightened clinical vigilance, especially when imaging findings and symptom progression suggest possible ischemia. This case exemplifies the importance of maintaining a broad differential diagnosis in adults with bowel obstruction. While in this case, initial imaging was suggestive of a Meckel’s diverticulum or a mesenteric cyst, imaging may not always be as definitive.

While Meckel’s diverticulum can have symptomatic mimickers such as mesenteric cysts, appendicitis, ileal/colonic diverticulitis, regional enteritis/colitis, enteric duplication cyst, and some types of intussusception, there are several means to narrow the diagnosis. Meckel’s diverticulum often has an antimesenteric location with communication with the bowel, more irregular mucosa, a persistent vitellointestinal artery arising from the superior mesenteric artery, and an inverted Meckel's diverticulum can have a central core of fat attenuation surrounded by a collar of soft-tissue attenuation; the “double target” sign on ultrasonography. Ruling out appendicitis, any involvement of other regions of bowel, any dilatation of the pelvicalyceal system, or any connection to the umbilicus is important, as those findings are suggestive of other pathologies [10].

Enteric duplication cysts (EDCs) and Meckel's diverticulum are differentiated pathologically by their location relative to the bowel mesentery, muscle wall structure, and communication with the intestinal lumen. EDCs typically lie on the mesenteric side, share a muscular wall with the intestine, and have two smooth muscle layers, whereas Meckel's diverticulum occurs on the antimesenteric border and is a true diverticulum. This is important because in this case, the pathology report showed no mucosa, but this is attributed to the fact that the Meckel's diverticulum was infarcted and necrosed, leading to loss of the mucosa layer.

In this case of a giant Meckel’s diverticulum, the initial imaging was suggestive of a mesenteric cyst or a Meckel’s diverticulum. A mesenteric cyst is often a well-circumscribed, unilocular, fluid-attenuated lesion that arises within the mesentery, often displacing bowel rather than arising from it, with no communication with the bowel lumen, and can grow to be quite large. The initial CT imaging showed a large, homogeneous cystic structure with displacement of adjacent bowel, which was more suggestive of a mesenteric cyst. A typical Meckel’s diverticulum is often smaller, with a tubular or saccular structure, wall thickening, and a possible connection to the small bowel. In the initial CT scan, the lesion appeared too large and cystic for a typical Meckel’s diverticulum, with no obvious bowel continuity, and the location was considered atypical for a normally sized Meckel’s diverticulum.

While Meckel's diverticulum is frequently omitted from the adult differential, it should be considered, especially when there is evidence of closed-loop obstruction, unexplained free fluid, or signs of ischemia without a clear etiology. Managing complicated Meckel's diverticulum requires surgery, and early intervention significantly improves outcomes. In this case, surgical management was delayed due to delayed diagnosis, and no early laboratory findings suggestive of necrosis, ischemia, or bowel obstruction, which allowed the diverticulum to create a bowel obstruction and become ischemic and necrosed.

## Conclusions

This case highlights the challenges of diagnosing giant Meckel's diverticulum. It also emphasizes the significance of early recognition, timely diagnosis, and emergent surgical intervention. Initially, the patient was thought to have a mesenteric cyst vs. Meckel’s diverticulum, which are both benign lesions. One of the main imaging diagnostic complications in this case that led to diagnostic delay is that a mesenteric cyst and a giant Meckel's diverticulum can appear very similar on imaging due to the fact that they both can appear as a well-circumscribed, unilocular, and fluid-attenuated lesion. A typical Meckel’s diverticulum would present as a smaller tubular or saccular structure; thus, it was not consistent with the radiological findings in this case. A delay in diagnosis led to ischemic complications, including obstruction and a gangrenous diverticulum; therefore, the purpose of this case report was to emphasize the importance of thoroughly investigating patients who present with symptoms that may seem minor, such as abdominal pain. Even though this is just one patient’s account, the clinical presentation, surgical intervention, and adjunctive medical course of treatment for symptomatic giant Meckel’s diverticulum can offer insights applicable to a broader patient population. Thus, clinicians should not disregard giant Meckel's diverticulum as one of the possible differential diagnoses in adults presenting with unexplained non-specific abdominal symptoms.
